# Retraction Note: ANGPTL4 functions as an oncogene through regulation of the ETV5/CDH5/AKT/MMP9 axis to promote angiogenesis in ovarian cancer

**DOI:** 10.1186/s13048-023-01108-2

**Published:** 2023-01-26

**Authors:** Yinping Liu, Rui Yang, Yan Zhang, Yaping Zhu, Wei Bao

**Affiliations:** 1 Qingpu Branch of Zhongshan Hospital, Fudan University, 1158 Gongyuandong Road, Qingpu District, 201700 Shanghai, P. R. China; 2 Department of Obstetrics and Gynecology, Shanghai General Hospital, Shanghai Jiaotong University School of Medicine, No. 85 Wujin Road, Hongkou, 200080 Shanghai, P. R. China; 3 Department of Obstetrics and Gynecology, Shanghai General Hospital, Shanghai Jiaotong University School of Medicine, No. 85 Wujin Road, Hongkou, 201620 Shanghai, P.R. China

**Retraction Note: J Ovarian Res 15, 131 (2022)**

10.1186/s13048-022-01060-7

The Editors-in-Chief have retracted this article. After publication, concerns were raised regarding the presented images and animal ethics. Specifically, in Fig. 2A, the images representing LV-shANGPTL4 #1 and #2 (24 h) appear to originate from the same sample (rotated 180°). In addition, the total tumour burden presented in Fig. 2D-G exceeds the recommended weight and volume limits for mouse xenograft experiments.

The authors have stated that the same image was used in Fig. 2A by mistake, and that the weight and size of the tumours mentioned in this paper were the sum of the size of all miliary and rice-like tumors in the mouse peritoneal cavity, not the size of a single tumor. However, the total tumour size and weight exceeded the humane endpoints prescribed in the ethics approval for the study. Therefore, the Editors-in-Chief have decided to retract this article due to lack of adherence to the journal's expected ethical standards.

All authors agree to this retraction.

